# A Direct Oral Anticoagulant-Refractory Unusually Large Thrombus Attached to the Implantable Cardioverter-Defibrillator in a Patient on Rivaroxaban: A Case Report

**DOI:** 10.7759/cureus.101462

**Published:** 2026-01-13

**Authors:** Xin Y Liu, Manas Bajpai, Bendan Brew, Amresh Gul, Voltaire Nadurata, Zahid Khan

**Affiliations:** 1 Cardiology, Bendigo Health, Bendigo, AUS; 2 General Practice, General Practice Clinic, Brisbane, AUS; 3 Cardiology, William Harvey Research Institute, Queen Mary University of London, London, GBR; 4 Cardiology, University of South Wales, Pontypridd, GBR; 5 Cardiology, University of Buckingham, London, GBR; 6 Cardiology, Barts Heart Centre, London, GBR

**Keywords:** cardiac implantable electrical devices, guideline-directed medical therapy, heart failure with reduced ejection fraction, new york heart association (nyha) class, subcutaneous implantable cardioverter defibrillators, sudden cardiac death (scd), systemic anticoagulation, therapeutic anticoagulation, thromboembolism drug therapy, thromboembolism icd

## Abstract

Implantable cardioverter-defibrillators (ICDs) are the established method for the primary prevention of cardiac arrest in patients at risk of sudden cardiac death due to life-threatening ventricular arrhythmias or in selected patients with heart failure with reduced ejection fraction. The transvenous ICD (TV-ICD) system has been the established therapy for several decades; however, the complication of intracardiac thrombi on transvenous leads is a known risk. We present a case of a 54-year-old gentleman with previously implanted TV-ICD for ischaemic cardiomyopathy, who presented to the outpatient department for a routine echocardiogram. The echocardiogram showed a large ICD-lead thrombus despite being compliant with his anticoagulation therapy of rivaroxaban. He denied any shortness of breath, chest pain, fever, or night sweats. His case was discussed at the multidisciplinary team meeting, and the outcome was to switch from rivaroxaban to warfarin and to repeat the echocardiogram in six months. He also underwent transoesophageal echocardiogram, confirming the presence of a large ICD thrombus, and stayed in hospital for 72 hours. This case underscores the significance of anticoagulation pharmacokinetics when managing ICD-lead thrombi and highlights the potentially catastrophic sequelae of thrombi in patients with implantable cardiac devices. The occurrence of large ICD or pacemaker thrombus in anticoagulated patients who remain compliant with therapy is less common, and the anticoagulation option should be carefully discussed. Pacing lead thrombosis is a recognised complication of implantable cardiac devices. The risk is lower in optimally anticoagulated patients, and the management of pacing lead thrombosis depends on the thrombus size. Patients with smaller thrombi are at lower risk of serious complications than those with larger thrombi. Patients with implanted cardiac devices should be considered for anticoagulation if clinically indicated. Patients with implanted cardiac devices already on anticoagulation pose a significant clinical challenge.

## Introduction

Implantable cardioverter-defibrillators (ICDs) are currently one of the most effective therapies in preventing sudden cardiac death (SCD) for both primary and secondary prevention. SCD represents the most common cause of death in high-income countries, thought to account for 15-20% of all-cause mortality and half of all cardiovascular deaths [1-7]. Multiple trials and guidelines have evaluated the role of ICDs in reducing total all-cause mortality, including SCD as a primary preventative strategy [1,8]. The strongest recommendation for ICD as primary prevention applies to patients with ischaemic cardiomyopathy, heart failure with reduced ejection fraction (HFrEF) ≤35% despite optimal guideline-directed medical therapies (GDMT), New York Heart Association (NYHA) functional class II/III, and more than one year of life expectancy [1]. The transvenous ICD (TV-ICD) system has been the conventional method of therapy for several decades, offering low-energy cardioversion, high-energy defibrillation, and pacing capabilities, including anti-tachycardia pacing, pause prevention, and post-defibrillation pacing [9,10]. Younger patients, on the other hand, may also be suitable for subcutaneous ICD (S-ICD) [10]. Patients with severely impaired left ventricular systolic function with broad QRS complexes may require cardiac resynchronised therapy, defibrillator or pacemaker, depending on their functional and clinical status, and these patients tend to benefit from TV-ICD.

However, a major concern of the TV-ICD system is the high incidence of both short and long-term complications. TV-ICD patients are at risk of developing lead-related complications, breakdown of insulation, infection, and thrombotic events, with mechanical and infective compromise increasing with time [11,12]. In particular, the risk of intracardiac thrombi on transvenous leads from cardiac implantable electrical devices (CIEDs) is a well-recognised complication, with a prevalence ranging from 0.6% to 3.5% [3-7]. There are substantial risks of ICD-lead thrombi, such as pulmonary embolism, cerebrovascular events, valvular regurgitation, and pacemaker dysfunction. Despite this, the optimal approach to management includes anticoagulation for smaller thrombi, whereas surgical excision and extraction may be required for larger thrombi [13]. Anticoagulation therapy without any obvious risk is not indicated in patients with ICDs. Several possible explanations for thrombi formation in patients with ICD include endothelial injury by long-term pacemaker presence, platelet aggregation after pacemaker implantation and hypercoagulable states such as pregnancy, heart failure, and malignancy.

The approach to management depends on infectious signs and symptoms and comorbidities, including hypercoagulable and arrhythmogenic disorders [12,13]. In most cases of reported pacemaker or ICD-lead thrombi, a risk-benefit analysis of intervention is required to decide between surgical or medical management in the first instance. This case presents the diagnostic dilemma of an incidental ICD-lead large thrombus in a relatively asymptomatic 54-year-old gentleman, already anti-coagulated on rivaroxaban for many years.

## Case presentation

A 54-year-old gentleman of Indigenous background known to the cardiac service at our health service was incidentally found to have a large ICD lead mass on his routine transthoracic echocardiogram (TTE), despite taking regular antiplatelet and anticoagulant therapy, including ticagrelor 90 mg twice daily and rivaroxaban 20 mg daily. The patient had a significant cardiac history, including paroxysmal atrial fibrillation (pAF), HFrEF, previous deep vein thrombosis, dyslipidaemia, and ischaemic cardiomyopathy. He suffered four previous myocardial infarctions between 2016 and 2020 and underwent six coronary angioplasties with stents to the left anterior descending artery (LAD) and the right coronary artery (RCA). His last myocardial infarction was in 2020, and he underwent percutaneous coronary intervention (PCI) with a drug-coated balloon to LAD in-stent restenosis and a stent to mid-distal LAD. A dual-chamber ICD was inserted in 2021 as a primary preventative therapy in view of his severely impaired left ventricle (LV) function despite optimal GDMT for six months. He was lost to follow-up from the cardiology clinic in 2023. However, he continued to be followed up in the pacing clinic and underwent a repeat echo due to episodes of ventricular tachycardia detected on his device interrogation. He had continued with an unusual combination of ticagrelor 90 mg twice daily and rivaroxaban 20 mg once daily since 2020.

**Figure 1:**
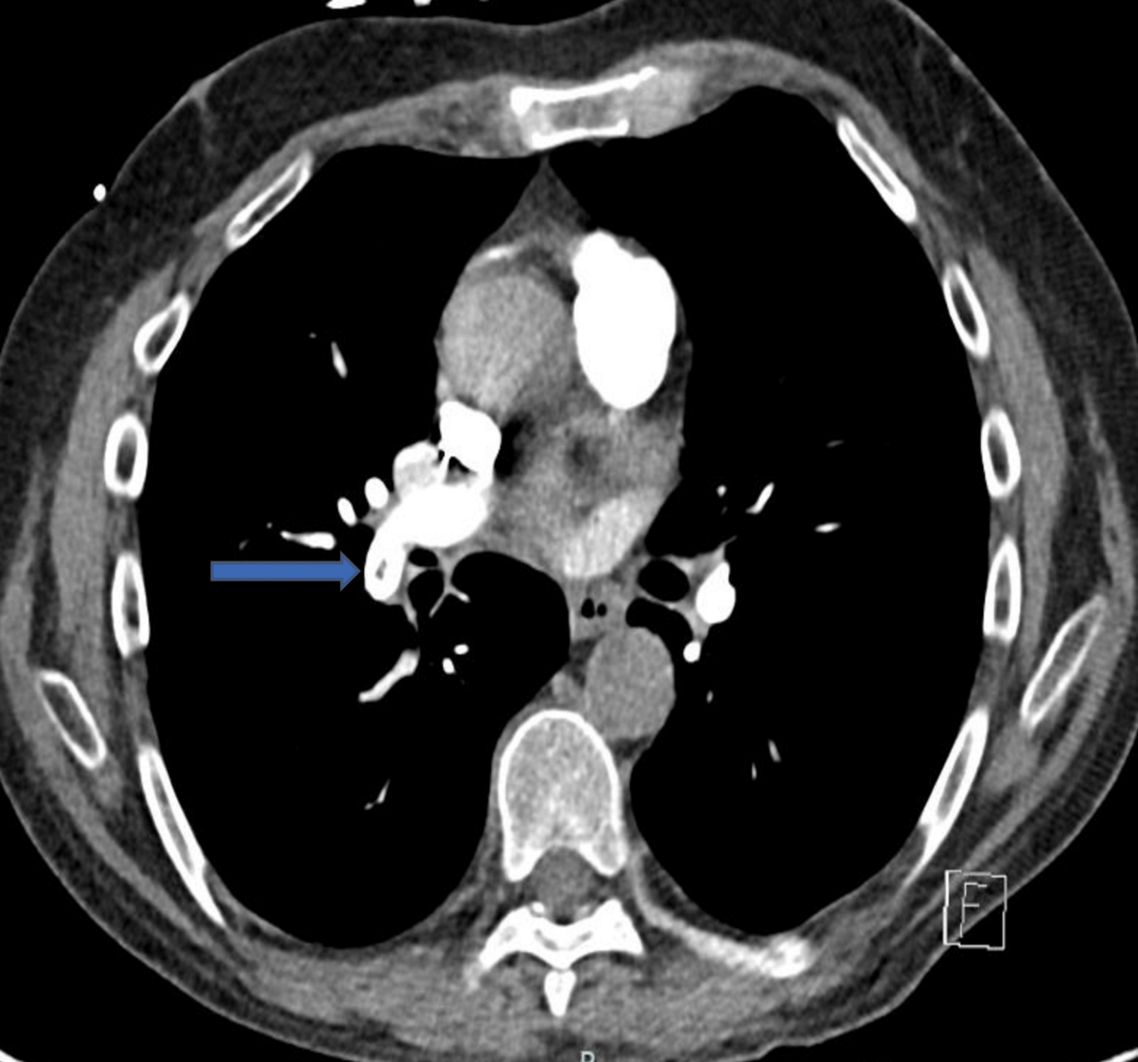
Right pulmonary artery embolism on computerised pulmonary angiogram (blue arrow).

Echocardiography revealed a large 25 x 16 mm mass attached to the ICD lead and mild tricuspid regurgitation (Videos 1, 2). The mass was suspicious for a vegetation or thrombus; however, as the patient was apyrexial, blood cultures were taken, but he was not commenced on antibiotics. He also underwent computerised pulmonary angiography (CTPA), revealing a few smaller defects consistent with small pulmonary embolism in the right pulmonary artery and right lower lobe pulmonary artery (Figure 1). At the time of admission, he only occasionally experienced mild, sharp chest pains but otherwise was asymptomatic. Blood cultures, Q fever, and Bartonella serology were all negative. Furthermore, he reported no medical history of fever, night sweats, or intravenous drug use and was screened to be negative for lupus anticoagulant antibody. He proceeded to an inpatient transoesophageal echocardiogram (TOE) to further characterise the identified ICD-lead mass. The TOE identified a large, calcified thrombus attached to the ICD lead, which prolapsed through the tricuspid valve during the cardiac cycle (Videos 3, 4). As the patient was already anticoagulated, there was a concern with continuing direct oral anticoagulant (DOAC) therapy, and given the size of the thrombus, the patient was referred to a multidisciplinary team meeting to discuss his management.

**Video 1:** Apical four-chamber transthoracic echocardiogram showing mild tricuspid regurgitation.

**Video 2:** Apical four-chamber transthoracic echocardiogram showing a large thrombus attached to the implantable cardioverter defibrillator lead.

**Video 3:** Transoesophageal echocardiogram showing thrombus attached to the implantable cardioverter defibrillator lead.

**Video 4:** Transoesophageal echocardiogram showing a large thrombus attached to the implantable cardioverter defibrillator lead.

The case was discussed at a tertiary cardiology and cardiothoracic team case conference. While it was unclear why both ticagrelor and rivaroxaban were used in combination, the recommendation to discontinue ticagrelor and commence warfarin was adopted in the patient’s management. The half-life of rivaroxaban, which is about nine hours in younger patients and 11-13 hours in elderly patients, was highlighted as a possible cause of thrombus formation outside the anticoagulant’s therapeutic window. The patient was subsequently commenced on slow loading of warfarin with a 5 mg dose initially and referred to a pathology service for regular international normalised ratio (INR) monitoring to titrate his warfarin dosing. He was also referred for an outpatient TTE in two months to assess the changes of the ICD-lead thrombus and the therapeutic effect of warfarin. His antiplatelet therapy was stopped, and he was advised to continue with warfarin till thrombus resolution. He can be considered for switching to apixaban 5 mg twice daily after the thrombus resolves. His INR at the time of discharge was 2.7, showing therapeutic anticoagulation. He remains under clinical follow-up and was advised to adhere strictly to medical therapy and anticoagulation.

## Discussion

The transvenous approach has been the mainstay of implanting endocardial leads for CIEDs, including pacemakers or ICDs, for five decades [1,2,10-12]. In the TV-ICD system, transvenous defibrillator or pacing leads are passed across the tricuspid valve, through the right atrium, and into the right ventricle to provide pacing and defibrillation capabilities. Alongside established risks such as infection, lead dysfunction, and lead damage, there is the risk of sterile intracardiac thrombi that can attach to leads [13-18]. The current literature likely underestimates the true prevalence of transvenous lead-related thrombosis, as cases are commonly identified incidentally at autopsy or in individuals referred for transvenous lead extractions or cardiac ablations during the perioperative period [14,19-21].

Various imaging modalities have been used to evaluate incidental intracardiac masses associated with transvenous leads [3-7]. The prevalence of transvenous lead masses in studies using intracardiac echocardiography (ICE) ranges from 0.6% to 3.5% in patients undergoing cardiac ablation [3,4]. However, 9% to 18.5% of individual patients undergoing transoesophageal echocardiography (TOE) were found to have lead thrombi [17,18]. However, characteristics such as thrombus size and location contributed to detection. Thrombi within the right atrium and proximal superior vena cava (SVC), with a size of <2 cm, may be more readily detected [21]. A recent study has further highlighted the underestimation of CIED-lead thrombus prevalence, with 48% of thrombi found on atrial leads and 33% on ventricular leads in autopsies [22]. As such, comparisons between imaging modalities may represent an insightful area of research to evaluate the accuracy and effectiveness of transvenous-lead mass identification [14].

There is limited literature on the prognosis of CIED implantation in patients with developing lead thrombi [16-22]. The prospective study by Chow et al. remains the sole study to date to examine the prevalence of ICD-lead thrombi specifically [23]. In this study, ICD-lead thrombi were identified by TOE in 25% of patients four months after implantation. Patients with and without ICD-lead thrombi differed in the use of anticoagulant and defibrillator lead insulation. ICD-lead thrombi were more common in patients not on anticoagulation, specifically warfarin (Coumadin), and those with polyurethane-insulated leads. The results of Chow et al.’s study encourage the pursuit of larger clinical trials to determine the merit of prophylactic anticoagulation [23].

Incidentally detected thrombi warrant clinical concern as literature has documented CIED-lead associated complications including pulmonary embolism (subclinical and clinical), lead dysfunction, stroke, pulmonary hypertension, and tricuspid inflow obstruction [7-11]. Given these risks and the varying prevalence across largely heterogeneous studies, future studies should adopt rigorous sampling methods, patient selection, and relevant comparisons across imaging modalities to facilitate careful consideration of management strategies for transvenous-lead thrombi.

No standard guidelines currently exist for the management of transvenous lead thrombi. Specifically, data about the presence of intracardiac thrombi during DOAC therapy in individuals with atrial fibrillation (AF) are scant. In documented cases, management was like ours, with differentiation between infectious and non-infectious aetiology of the transvenous-lead mass made in the first instance. The confirmation of thrombotic aetiology was followed by discontinuation of DOAC and commencement of warfarin, with complete resolution of the thrombi within four to six weeks of therapeutic warfarin treatment [13,14,24]. Additionally, medical management of transvenous-lead thrombi primarily focuses on vitamin K antagonists, and the long-term continuation of therapy is largely determined by thromboembolic risk.

Furthermore, patients with AF and CIEDs have been found to have more than eight times the likelihood of developing CIED-associated clots than patients without AF [18]. The intensification of anticoagulation or antiplatelet therapy may be beneficial for preventing embolic sequelae by shrinking or resolving the thrombi [18]. In cases where (1) the transvenous lead thrombi caused lead dysfunction, (2) anticoagulation could not be started, and (3) the risk of embolism or acute occlusion of the right ventricular outflow tract was imminent, surgical management with lead extraction and replacement or thrombectomy was adopted [7,12,16]. Additionally, the European Heart Rhythm Association 2020 consensus recommended considering open lead extraction for thrombi >2 cm [16].

A retrospective study recently found that routine TTE identified mobile echo-densities in 1.6% of patients with CIEDs without clinical suspicion of vegetations during a mean 43-month follow-up period [25]. Conservative management was adopted in asymptomatic patients [25]. However, we postulate that irrespective of symptoms, management should be patient-centred and consider the various factors that predispose to thrombus formation.

Of particular interest is the mechanism underlying DOAC-refractory thrombus formation in our case. Current literature postulates that lead-dwell time is a major risk factor for lead-induced thrombus formation, with the long-term residence of a pacemaker or defibrillator serving as a nidus for thrombus formation and contributing to a continuous inflammatory and fibrotic response [11,14,15]. Furthermore, conditions such as heart failure, renal failure, antiphospholipid syndrome, AF, and malignancies may predispose to thrombosis [14,15]. In our case, we speculated that the patient’s hypercoagulable state associated with HFrEF and AF and the pharmacokinetics of rivaroxaban collectively predisposed him to the ICD-lead thrombus formation. It is well documented that the half-life of rivaroxaban ranges from eight to nine hours, potentially leaving a subtherapeutic window [26]. Interestingly, breakthrough ischaemic strokes during DOAC therapy in patients with non-valvular AF have been documented [26]. While studies have not commented on the relationship between DOAC half-life and the possibility of refractory thrombi formation, competing stroke mechanisms such as large vessel atherosclerosis, cardio-embolism, and small-vessel occlusion have been suggested to contribute to anticoagulant treatment failure [27]. While randomised trials have found that the efficacy and safety of rivaroxaban for treating left ventricular thrombus are non-inferior to warfarin, the presence of DOAC-refractory thrombosis warrants further research to explore the underlying pathogenesis and identify optimal treatment and preventive therapies for transvenous-lead-associated thrombi [28]. A meta-analysis comprising seven randomised controlled trials (RCTs) found that DOACs compared to warfarin in patients with AF and a prior stroke were associated with a 15% reduced risk of recurrent stroke or systemic embolism and a 47% reduced risk of intracranial haemorrhage [27].

The current literature suggests that a risk-benefit analysis is essential to deciding between surgical and medical interventions. In cases of asymptomatic CIED-lead-associated thrombi or masses, conservative observation may be adopted; however, lead-dwell time is an intrinsic factor that precipitates thrombus formation, and thus, prophylactic anticoagulation and routine imaging should be considered for long-term CIED management [16,29].

## Conclusions

This report presents an interesting example of the development of an ICD-lead thrombus during rivaroxaban and ticagrelor combination therapy. It highlights the role of collaborative communication among larger tertiary hospitals in guiding management towards effective comprehensive care, as well as the limitations of current literature in establishing a guide for management. However, while the current data are of poor quality, warfarin could be an effective alternative for the management of DOAC-refractory thrombus.
